# The Sanitation Hygiene Infant Nutrition Efficacy (SHINE) Trial: Rationale, Design, and Methods

**DOI:** 10.1093/cid/civ844

**Published:** 2015-11-11

**Authors:** The Sanitation Hygiene Infant Nutrition Efficacy (SHINE) Trial Team

**Affiliations:** Zvitambo Institute for Maternal and Child Health Research, and Ministry of Health and Child Care, Harare, Zimbabwe; Johns Hopkins Bloomberg School of Public Health, Baltimore, Maryland; Queen Mary University of London, United Kingdom; Cornell University, Ithaca, New York; University of British Columbia, Vancouver, Canada; University of Michigan, Ann Arbor; Middlebury College, Vermont; George Washington University, Washington D.C.

**Keywords:** sanitation, hygiene, stunting, anemia, environmental enteric dysfunction

## Abstract

Child stunting and anemia are intractable public health problems in developing countries and have profound short- and long-term consequences. The Sanitation Hygiene Infant Nutrition Efficacy (SHINE) trial is motivated by the premise that environmental enteric dysfunction (EED) is a major underlying cause of both stunting and anemia, that chronic inflammation is the central characteristic of EED mediating these adverse effects, and that EED is primarily caused by high fecal ingestion due to living in conditions of poor water, sanitation, and hygiene (WASH). SHINE is a proof-of-concept, 2 × 2 factorial, cluster-randomized, community-based trial in 2 rural districts of Zimbabwe that will test the independent and combined effects of protecting babies from fecal ingestion (factor 1, operationalized through a WASH intervention) and optimizing nutritional adequacy of infant diet (factor 2, operationalized through an infant and young child feeding [IYCF] intervention) on length and hemoglobin at 18 months of age. Within SHINE we will measure 2 causal pathways. The program impact pathway comprises the series of processes and behaviors linking implementation of the interventions with the 2 child health primary outcomes; it will be modeled using measures of fidelity of intervention delivery and household uptake of promoted behaviors and practices. We will also measure a range of household and individual characteristics, social interactions, and maternal capabilities for childcare, which we hypothesize will explain heterogeneity along these pathways. The biomedical pathway comprises the infant biologic responses to the WASH and IYCF interventions that ultimately result in attained stature and hemoglobin concentration at 18 months of age; it will be elucidated by measuring biomarkers of intestinal structure and function (inflammation, regeneration, absorption, and permeability); microbial translocation; systemic inflammation; and hormonal determinants of growth and anemia among a subgroup of infants enrolled in an EED substudy. This article describes the rationale, design, and methods underlying the SHINE trial.

***Clinical Trials Registration.*** NCT01824940.

Globally, stunting affects 26% (165 million) of children aged <5 years, underlies 15%–17% of their mortality [1], and leads to long-term cognitive deficits, fewer years and poorer performance in school, lower adult economic productivity, and a higher risk that their own children will also be stunted, perpetuating the problem into future generations. Stunting begins antenatally and peaks at 18–24 months of postnatal life, when mean length-for-age *z* score (LAZ) is approximately −2.0 among children living in Africa and Asia [2]. Inadequate diet and recurrent illness (especially diarrhea) have been the 2 most commonly implicated causes of stunting, and therefore the focus of most research designed to inform prevention strategies.

Dietary studies have tested a myriad of nutrient-dense foods including high-energy milks [3, 4], nutrient-dense porridges [5–8], and bacterial-resistant, micronutrient-fortified lipid pastes [9–11]. Studies have also tested different combinations of nutrition education without provision of food [12, 13], food without education [3, 5, 8], and food given together with education [14, 15]. Behavior change strategies have been undertaken to persuade mothers to feed their children more responsively to hunger cues in a physically comfortable and psychologically supportive environment [16]. A systematic review of 38 of the best studies revealed that children who received these interventions grew 0–1.7 cm taller (LAZ, 0.0–0.64) by 12–24 months compared with control children [17], indicating that the growth effect of the most efficacious dietary interventions is equivalent to about one-third of the average deficit experienced by Asian and African children (LAZ score approximately −2.0). This suggests that, at best, optimizing infant diet can solve about one-third of the stunting problem.

Studies estimating the effect of diarrhea on linear growth have reached inconsistent conclusions. In a pooled analysis of 9 studies that together collected diarrhea and growth data on 1393 children, the odds of stunting at 24 months increased multiplicatively by 2.5% per episode of diarrhea, and 25% of all stunting among 24-month-old children was attributable to having 5 or more episodes of diarrhea in the first 2 years of life [17]. In other studies, the observed effect of diarrhea on long-term linear growth was small or absent because children grew at higher-than-average “catch-up” growth rates between illness episodes [18, 19]. Most recently, an analysis of 7 longitudinal cohort studies of children aged <2 years, conducted in 4 low-income countries, estimated that the average child's diarrhea burden (46 days with diarrhea between birth and 2 years) was associated with a 0.38-cm height deficit at 2 years of age [18]. Because a change in LAZ of 1.0 at 2 years is equivalent to approximately 3 cm [19], their analysis suggests that the growth effect of reducing diarrhea during the first 2 years of life from the average burden currently experienced in low-income countries to zero is a statistically significant but clinically modest LAZ score of approximately 0.13, or about 7% (one-fifteenth) of the average height deficit of 2-year-old Asian and African children.

Like stunting, childhood anemia is a major public health problem in Africa and Asia [1] and a primary cause of cognitive and behavioral developmental delay throughout childhood and adolescence [20]. The 2010 Global Burden of Disease ranks iron deficiency anemia as the 15th leading cause of lost disability-adjusted life-years globally [21]. Anemia becomes prevalent in the second half of infancy and peaks between 12 and 24 months of age [22], affecting well over half of all children in Africa and Asia [23]. Iron deficiency causes about half of the anemia among children under 2 [24]. Accordingly, increasing iron intake by young children has been the focus of most research and public health programming, and, in randomized trials, iron supplements or iron-fortified foods reduce anemia in young children by about 37%–62% [25], leaving a substantial portion of child anemia unaddressed.

The Sanitation Hygiene Infant Nutrition Efficacy (SHINE) trial is motivated by a 2-part premise [26]:

- A major cause of child stunting and anemia is environmental enteric dysfunction (EED). EED is a subclinical disorder of the small intestine, which is virtually ubiquitous among asymptomatic people living in low-income settings throughout the world [27–38]. EED is characterized by increased permeability, which facilitates microbial translocation into the systemic circulation and triggers chronic immune activation [39, 40].
- The primary cause of EED is infant ingestion of fecal microbes due to living in conditions of poor quality and quantity of water, sanitation, and hygiene (WASH).

## EED Develops During Infancy, Is Strongly Associated With Poor Growth, and May Cause Anemia

Studies of stillborn fetuses have established that EED is not present at birth [41]. Seminal research conducted over 4 decades in The Gambia demonstrated that EED develops during early infancy and is strongly associated with poor growth [42–52]. These studies showed that stunting was not explained by diarrheal burden or inadequate dietary quantity or quality [45, 46], but was associated with gut permeability (assessed by the lactulose-mannitol [LM] test) and immune activation (assessed by plasma concentrations of immunoglobulin G [IgG] and endotoxin core antibody) [51]. Among children studied longitudinally from 2 to 15 months of age, these biomarkers increased as LAZ plummeted and, in a regression model, IgG and IgG endotoxin core antibody concentrations explained 51% of the variability in linear growth during this age interval [48]. In a more recent study (The Interactions of Malnutrition & Enteric Infections: Consequences for Child Health and Development), in which 8 longitudinal cohorts of children were followed from birth to 2 years, an index of 3 fecal biomarkers of intestinal inflammation (neopterin, myeloperoxidase, and α-1 antitrypsin) predicted change in LAZ over the subsequent 6 months [53]. Finally, in a retrospective case-control study in Zimbabwe, cases (children who were stunted at 18 months of age) had higher concentrations of inflammatory markers (serum C-reactive protein and α-1 acid glycoprotein) and lower concentrations of plasma insulin-like growth factor 1 (IGF-1) between 6 weeks and 12 months of age compared to controls (children with normal LAZ at 18 months) [24]. These findings suggested that growth hormone resistance, characterized by inflammatory suppression of plasma IGF-1 (an already well-established mechanism of linear growth failure in children with juvenile idiopathic arthritis [54] and Crohn disease [55]) may also underlie stunting among otherwise healthy children living in resource-constrained environments.

The chronic immune activation that is characteristic of EED also may cause anemia through 2 possible pathways. First, elevated proinflammatory cytokines trigger increased hepatic synthesis of hepcidin, which suppresses iron absorption and utilization [56, 57], leading to iron deficiency anemia [58]. Second, cytokines may act directly on differentiation of bone marrow stem cells, suppressing erythropoiesis and leading to anemia of inflammation [59].

## Conditions of Poor WASH Are Associated With EED in Humans

Evidence linking poor WASH with EED in humans is limited to observational studies. Among 3- to 5-year-old children in Malawi, those in households with latrines and a greater quantity of water had lower (more normal) LM ratios, and children who washed their hands more often or washed their hands with soap had a more normal sucrose-lactulose ratio (an indicator of gastric permeability) [60]. Similarly, Bangladeshi children living in clean compared to dirty households (based on water quality, facilities for hand washing, and sanitation) had lower urinary LM ratios and lower plasma IgG concentrations [61]. A small intestinal biopsy study of adult volunteers in Lusaka, Zambia and Soweto, South Africa found that all the Lusaka residents but none of the Soweto residents had morphometric changes of EED; at the time of the study, sanitation coverage was 59% in Lusaka [62] and 99.9% in Soweto [63].

## WASH May Be More Strongly Associated With Child Stunting Than With Diarrhea

Numerous observational studies have reported a strong association between the level of environmental WASH and child linear growth [61, 64–70]; indeed, in some, conditions of poor WASH were more strongly associated with stunting than with diarrhea. Esrey analyzed Demographic Health Survey data on nearly 12 000 rural children from 8 developing countries and found that neither improved sanitation nor water was significantly associated with diarrhea, but both were associated with increases of 0.05–0.6 in LAZ [64]. In a prospective cohort study evaluating the child health benefits of water and sanitation services in Peru, diarrhea prevalence explained only 16% of children's height deficit at 2 years of age, while differences in water and sanitation services explained 40% of this deficit [22]. These findings imply that the primary pathway linking WASH to growth may be largely independent of diarrhea.

## Among WASH Interventions, Sanitation May Be Central

Adequate sanitation may be a particularly important requirement for normal growth. In the Esrey analysis, the association with growth was stronger for sanitation than water services [64]. In the Peru study, the growth effects of improved water source were magnified when concurrent with improved sanitation [22]. In a large ecological analysis of data from India (where about half the population lacks sanitation facilities), Spears estimated that differences in open defecation rates explained 35%–55% of the average difference in stunting between districts [71]. This apparent importance of sanitation could partly explain the relatively small magnitude of impact of WASH on linear growth (LAZ, +0.08) observed in a recent systematic review of 5 randomized controlled trials that tested improved drinking water quality and/or hand washing, but did not include improved sanitation [72]. Finally, an econometric analysis of data from 26 low-income countries estimated that a 15-minute reduction in walking time to collect water (an indicator of water access) is associated with an increase in height-for-age *z* score (HAZ) of 0.26 and that this effect was stronger among households with sanitation (HAZ, +0.35) vs without sanitation (HAZ, +0.21) [73].

## Unhygienic Living Conditions Restrict Growth in Animals and This Effect Is Mediated by Inflammation

Controlled trials in animal husbandry provide strong evidence that poor WASH restricts animal growth and that this effect is mediated by inflammation. In a factorial trial in which chicks were randomized to live either in cages with accumulated feces, dust, and dander (“dirty chicks”) or steam-cleaned cages (“clean chicks”), and to receive either antibiotics or no antibiotics, the clean chicks grew better than dirty chicks. However, the dirty chicks receiving antibiotics grew as well as the clean chicks not receiving antibiotics; overall, antibiotics had no growth effect in clean chicks [74]. The poor growth in dirty chicks was accompanied by high plasma concentrations of interleukin 1 (IL-1), a key proinflammatory cytokine. Levels of IL-1 were significantly lower in clean chicks and in dirty chicks fed antibiotics, suggesting that antibiotics may promote growth by reducing immune stress.

## High Exposure to Fecal Microbes May Cause EED

The pathogenesis of EED, similar to other inflammatory intestinal diseases, results from unrestrained enteric T-cell activation: biopsy studies of the human small intestine demonstrate rapid development of crypt hyperplasia, villous atrophy, and mucosal destruction in response to chronic T-cell stimulation [75–77]. However, unlike celiac disease, in which *abnormal T cells* are hyperreactive to a *normal exposure* (ie, dietary gluten [78]), EED may be the result of *normal T-cells* appropriately reactive to *excessive exposure* of fecal bacteria [79]. Bacterial colonization has long been recognized to influence intestinal morphology. In an animal study from 1961 [80], germ-free guinea pigs were found to have an immature intestinal structure. Controlled introduction of bacteria to germ-free animals led to a progressive change in mucosal architecture, but to a lesser degree than guinea pigs reared in conventional conditions. These animals, exposed to environmental bacteria and other insults, had substantial villous shortening and thickening and crypt elongation. It is therefore plausible that exposure to large quantities of fecal microbes among children living in conditions of poor WASH drives the profound mucosal changes characteristic of EED. This may be particularly true in the context of hypochlorhydria, where gastric barrier function is reduced, leading to extensive colonization of the small intestine. We hypothesize that, while only pathogenic bacteria cause diarrhea, excessive exposure to all fecal microbes (including those from healthy people and animals) may contribute to EED. If true, EED prevention may require more comprehensive interventions than those designed to reduce diarrhea.

## Summarizing the Background and Linking It to the SHINE Trial Design

Childhood stunting and anemia are 2 highly prevalent, frequently overlapping problems among children aged <2 years in Africa and Asia, which result in immediate and long-term morbidity and cognitive deficits, lost human capacity, and reduced adult economic productivity. Moreover, both problems are only partially responsive to current public health interventions. SHINE is motivated by the premise that EED is a major underlying cause of stunting and anemia, that chronic inflammation is the central characteristic of EED mediating these adverse effects, and that EED is primarily caused by high fecal ingestion due to living conditions of poor WASH. SHINE will also investigate whether these hypothesized effects of improved WASH will be additive to those of concurrently improved infant feeding in reducing child stunting and anemia. Accordingly, SHINE is designed as a proof-of-concept, 2 × 2 factorial trial to assess the independent and combined effects of protecting babies from fecal ingestion (factor 1, operationalized through a WASH intervention) and optimizing nutritional adequacy of infant diet (factor 2, operationalized through an infant and young child feeding [IYCF] intervention) on length and hemoglobin at 18 months of age. Within SHINE we are measuring 2 causal pathways. The program impact pathway comprises the series of processes and behaviors linking implementation of the interventions with the 2 child health primary outcomes; it will be modeled using measures of fidelity of intervention delivery and household uptake of promoted behaviors and practices [81]. We are also measuring a range of household and individual characteristics, social interactions, and maternal capabilities for childcare [82], which we hypothesize will explain heterogeneity along these pathways. The biomedical pathway comprises the infant biologic responses to the WASH and IYCF interventions that ultimately result in attained stature and hemoglobin concentration at 18 months of age; it will be elucidated by measuring biomarkers of intestinal structure and function (inflammation, regeneration, absorption, and permeability); microbial translocation; systemic inflammation; and hormonal determinants of growth and anemia among a subgroup of infants enrolled in an EED substudy [83].

The WASH Benefits Trials in Bangladesh and Kenya [84] are ongoing trials similar to SHINE in the interventions being tested, outcomes measured, and research questions addressed. Regular meetings between the trial groups have enabled the sharing of preliminary findings and alignment of methods and outcomes, to facilitate comparison of trial results and enhance the joint contributions of the trials to global health.

## STUDY DESIGN OVERVIEW

The SHINE trial addresses one primary objective and multiple secondary objectives (Table 1). The 4 treatment arms of the trial are described in Table 2.

**Table 1.** Objectives of the Sanitation Hygiene Infant Nutrition Efficacy (SHINE) Trial

Primary objectives
To determine the independent and combined effects of improved household WASH and improved IYCF on length and hemoglobin concentration among children at 18 mo of age who are born to HIV-negative women in rural Zimbabwe.
Secondary objectives^a^
To examine the effects of the 2 randomized interventions (WASH and IYCF) on stunting (LAZ <−2) and anemia (hemoglobin <105 g/L) among children at 18 mo of age who are born to HIV-negative women in rural Zimbabwe.
To examine differential effects on length and hemoglobin concentration at 18 mo of the 2 randomized interventions (WASH and IYCF) in these prespecified subgroups: Children born to HIV-positive compared to HIV-negative mothers Male and female children Subgroups formed by categorizing household wealth, household distance from a water point, and maternal capabilities.
To examine the independent and combined effects of the 2 randomized interventions on body weight, mid-upper arm circumference and head circumference at 18 mo, and on all anthropometric measures at intermediate ages (1, 3, 6, and 12 mo).
To describe the PIP linking implementation of each randomized intervention (WASH and IYCF) with length and hemoglobin concentrations by assessing: The quality of VHW training and supervision VHW capacity, defined as a composite of attained knowledge, goal-setting capacity, and achieved performance; Fidelity of intervention implementation, defined as degree of conformance with protocol specifications for both VHW and mother; Attained maternal knowledge and skills assessed by questionnaire and observation; Uptake or adoption of promoted behaviors by mothers and their households assessed by questionnaire and observation.
To assess potential effect modifiers along the PIP: Individual VHW characteristics (age, time in post, intrinsic, and extrinsic motivational characteristics); Maternal capabilities, defined by a woman's physical and mental health, stress, time allocation, maternal self-efficacy, and autonomy; Household socioeconomic status; Intervention complexity (WASH and IYCF implemented together vs each intervention implemented alone); For the WASH intervention only, access to water, defined as distance to water in each season of the year.
To describe the prevalence of exclusive breastfeeding among all infants enrolled in the trial by maternal/infant HIV status.
To evaluate the effect of the IYCF intervention on uptake of improved infant feeding practices by maternal/infant HIV status, specifically: Infant diet quality as assessed by World Health Organization IYCF indicators Infant nutrient intake from complementary foods assessed by 24-h dietary recall Appropriate use of Nutributter from 6 to 18 mo
To evaluate the effect of the WASH intervention on the 5 key behaviors it promotes by maternal/infant HIV status: Proper disposal of animal and human feces Handwashing with soap after fecal contact Point-of-use chlorination of drinking water Protecting children from ingestion of dirt and feces Feeding baby freshly prepared foods, or reheating leftover food
To elucidate the biological pathways linking WASH and IYCF with linear growth and hemoglobin concentration by measuring domains of EED, stratified by maternal/infant HIV status^b^: The composition and function of the infant intestinal microbiota; Intestinal permeability and absorptive capacity (assessed by LM ratio urine test), inflammation (assessed by fecal myeloperoxidase, α-1-antitrypsin and neopterin), epithelial damage (assessed by plasma I-FABP), and regeneration (assessed by fecal REG1B); Microbial translocation (assessed by plasma soluble CD14 and soluble CD163); Systemic inflammation (assessed by LPS, EndoCAb, plasma soluble CD14, and soluble CD163); Two hormonal responses to immune activation (IGF-1 and hepcidin); Hemoglobin concentration, sTFR, and hepcidin.
To measure the impact of the 2 randomized interventions (WASH and IYCF) on incidence, prevalence, and severity of diarrheal disease in infants, stratified by maternal/infant HIV status.
To model the relative contributions of diarrheal disease and EED in mediating the effects of improved WASH on child length and hemoglobin concentrations, stratified by maternal/infant HIV status.
To measure the strength of association between severity of maternal EED and systemic inflammation during pregnancy with the risk of 6 adverse birth outcomes (miscarriage, stillbirth, premature delivery, fetal stunting, low birth weight and neonatal death), stratified by maternal HIV status.
To measure the strength of association between other potential causes of stunting and anemia (other than poor WASH or IYCF) with linear growth and hemoglobin: Maternal schistosomiasis infection during pregnancy; Maternal HIV infection together with adherence to antiretroviral and cotrimoxazole regimens during pregnancy and lactation; Infant HIV infection or exposure, together with adherence to antiretroviral and/or cotrimoxazole regimens; Exposure to dietary mycotoxin contamination by the mother during pregnancy and lactation, and by the infant during complementary feeding.

Abbreviations: AGP, α-1 acid glycoprotein; CRP, C-reactive protein; EED, environmental enteric dysfunction; EndoCAb, Endotoxin core antibiody; HIV, human immunodeficiency virus; I-FABP, intestinal fatty acid binding protein; IGF-1, insulin-like growth factor 1; IYCF, infant and young child feeding; LAZ, length-for-age *z* score; LM, lactulose-mannitol; LPS, lipololysaccharide; PIP, program impact pathway; REG1B, regenerating protein 1B; SHINE, Sanitation Hygiene Infant Nutrition Efficacy; sTFR, soluble transferrin receptor; VHW, village health worker; WASH, water, sanitation, and hygiene.

^a^ A complete list of trial secondary endpoints is prespecified in the SHINE Statistical Analysis Plan.

^b^ The final choice of biomarkers to define each domain of EED may change depending on emerging data.

**Table 2.** Randomized Arms in the Sanitation Hygiene Infant Nutrition Efficacy (SHINE) Trial

Standard of Care:^a^ Exclusive breastfeeding promotion for all infants, birth to 6 mo Strengthened PMTCT services Strengthened village health worker system	WASH: Standard-of-care interventions Provide household ventilated pit latrine, Tippy Taps, monthly liquid soap, water treatment solution, and protective play space Provide interpersonal communication interventions promoting feces disposal in a latrine, handwashing with soap, drinking water treatment, hygienic weaning, food preparation, and preventing babies from putting dirt and animal feces in their mouths.
IYCF:^a^ Standard of care interventions Provide 20 g/d Nutributter from 6–18 mo Provide interpersonal communication interventions promoting optimal use of locally available foods for complementary feeding after 6 mo, continued breastfeeding, and feeding during illness.	Sanitation/Hygiene AND Nutrition: Standard of care interventions All WASH interventions All IYCF interventions

Abbreviations: IYCF, infant and young child feeding; PMTCT, prevention of mother-to-child transmission of HIV; WASH, water, sanitation, and hygiene.

^a^ Women in standard-of-care and IYCF arms are provided with a latrine at the end of the trial.

Clusters are defined as the catchment area of 1–4 community-based village health workers (VHWs) of the Zimbabwe Ministry of Health and Child Care (MoHCC). More than 5000 pregnant women were identified in a surveillance system of early pregnancy detection using urine pregnancy tests and enrolled into the trial between November 2012 and March 2015. All enrolled women receive the same number (15) of visits from their VHW between enrollment and 18 months postpartum, during which standard-of-care (SOC) primary healthcare messages are delivered. During some of these 15 visits, VHWs in clusters randomized to WASH, IYCF, and WASH + IYCF also deliver lessons specific to their arm (Table 1 of Supplementary Appendix) so that women in active arms receive more information than women in the SOC arm. Each randomized intervention comprises 5 core modules, focused on a key message and delivered to the mother at a specified relevant fetal or infant age. A separate team of research staff employed by the trial make data collection home visits at baseline (approximately 14 weeks' gestation), at 32 weeks’ gestation, and at 1, 3, 6, 12, and 18 months postpartum to measure fidelity of intervention delivery, uptake of the interventions, and trial outcomes (Table 3). Women who move from their primary residence (their home at the time of recruitment) are not followed up at interim data collection visits, but extensive effort is made to trace and follow all women residing anywhere within Zimbabwe at the 18-month visit to collect trial endpoints for the intent-to-treat analyses.

**Table 3.** Interview, Observation, Measurement, Test Administration, Health Record Transcription, and Biologic Specimen Collection According to Fetal/Infant Age

Data Collected	Research Visit							
	Antenatal			Postnatal, mo				
	Baseline	32 wk	Birth	1	3	6	12	18
Household composition, socioeconomic status	I					I		I
IYCF and WASH knowledge and practices	I/O			I/O	I/O	I/O	I/O	I/O
Maternal factors								
Antenatal care, pregnancy exposures, PMTCT	I	I		I				
Sick clinic visits and hospitalizations	I	I		I	I	I	I	I
Maternal capabilities	I					I		I
Maternal depression		T				T	T	
Height	M							
Weight, mid-upper arm circumference	M	M		M	M	M	M	M
Blood pressure	T	T						
Biologic specimen collection, testing, and archival								
Blood: HIV, CD4 (baseline), Hb, EED panel in substudies^a^	All	All						All
Urine: Pre- and post-LM; spot for urinalysis/schistosomiasis^b^	All	All-spot						
Stool: EED panel in substudies	All			EED				
Saliva: cortisol	All							
Infant factors								
Delivery date, time, mode, place, complications, care, sex			R					
Weight, length, head circumference			R	M	M	M	M	M
Immunizations and vitamin A			R	R/I	R/I	R/I	R/I	R/I
7-day and long-term morbidity, sick clinic visits, hospitalizations				I	I	I	I	I
Biologic specimen collection, testing, and archival								
Blood: EED panel, HIV DNA PCR, Hb^c^				EED	EED	EED	EED	All
Urine: Pre- and post-LM test					EED	EED	EED	EED
Stool: EED panel				EED	EED	EED	EED	EED
Saliva					EED	EED	EED	EED

For specimen collections, All refers to all women and infants enrolled in the trial; EED refers to mother–infant dyads selected for EED substudy.

Abbreviations: EED, environmental enteric dysfunction; Hb, hemoglobin; HIV, human immunodeficiency virus; I, interview; IYCF, infant and young child feeding; LM, lactulose-mannitol; M, measurement; O, observation; PCR, polymerase chain reaction; PMTCT, prevention of mother-to-child transmission of HIV; R, transcription from medical record; T, test; WASH, water, sanitation, and hygiene.

^a^ EED Panel includes assays which assess causal pathways of EED and anemia. Planned assays are listed below though final choice of biomarkers may change depending on emerging data. Blood/Serum/Plasma: α-1 acid glycoprotein; C-reactive protein; intestinal fatty acid binding protein; insulin-like growth factor 1; activation levels of T-cells by flow cytometry; soluble transferrin receptor; hemoglobin; Urine: lactulose-mannitol ratio; Feces: myeloperoxidase, α-1-antitrypsin; neopterin; regenerating protein 1B; composition and function of intestinal microbiota.

^b^ A single-void urine sample (spot sample) is collected from all women at baseline and at 32 gestational weeks for urinalysis using Multistix test strips in the homestead and for urinary microscopy for ova of *Schistosoma hematobium* in the field laboratory. At baseline, an LM test is additionally conducted in all women. Prior to ingestion of LM solution, a pre-LM single-void urine sample is collected to measure baseline mannitol by mass spectrometry; after ingestion of LM solution, all urine is collected over a 2-hour period (LM urine), preserved with chlorhexidine and measured, then aliquots frozen at −80°C for subsequent analysis of lactulose and mannitol recovery by mass spectrometry.

^c^ Hemoglobin is not measured at 1 month of age.

## DEVELOPMENT OF THE INTERVENTIONS

The underlying biologic mechanisms of child stunting are poorly understood. For this reason, the hypothesized causal pathway was the primary principle guiding development of the 2 interventions designed to optimize the 2 biologic objectives for enrolled children between birth and 18 months of age (WASH intervention: children are protected from ingesting human and animal fecal microbes; IYCF intervention: children consume a nutritionally adequate diet). As much as possible, we chose messages, commodities, and delivery systems that could be economically sustainable at scale, but we prioritized potential biological efficacy over cost.

SHINE interventions were based on formative research (detailed in companion articles [85, 86]) and incorporated adult education evidence-based behavior change strategies, including motivational interviewing, repetition of key messages, elicitation of emotional triggers, culturally adapted visual aids and stories, interactive games, and respect for the circumstances and autonomy of participants [87–90]. Interventions target the mother, but all household members are encouraged to participate.

We chose to implement interventions through the existing MoHCC VHWs rather than creating a separate intervention delivery system. This decision was motivated partly by Zimbabwe's historically strong community health system, but primarily taken because VHWs (who are nominated by and live in the communities they serve) could achieve the greatest coverage and acceptability within these rural communities. Beginning in 2010, vacant VHW posts were filled (total staffing = 180 per district, 360 VHWs in the trial area) and new VHWs completed the standard MoHCC 5-month training program (5 weeks residential didactic sessions interspersed with on-the-job training) [62]. VHWs were trained in SHINE-specific modules in separate groups according to the randomized allocation of the cluster in which they live and work. Residential training in MoHCC district-level training facilities lasted 20 days for SOC, 30 days for WASH, 32 days for IYCF, and 35 days for WASH + IYCF. Throughout trial implementation, VHWs receive supportive supervision from intervention-specific SHINE nurses at least monthly. VHWs receive a $14 monthly allowance from the MoHCC. To compensate them for the additional time they spend on SHINE activities, the trial provides a grocery basket every 3 months and modest monetary performance-based incentives.

All interventions (including latrine construction) were pilot-tested in a district adjacent to the study area among 40 households with children <18 months. VHWs in the pilot district were trained to implement the study to assess the training requirements and support needs of this cadre before training the 360 VHWs in the SHINE districts.

### Standard-of-Care Intervention

VHWs are trained through the MoHCC curriculum to respond to a broad spectrum of health problems. The curriculum instructs VHWs to visit pregnant women and infants frequently, but the precise content or purpose of each visit is not specified. Consequently, the SHINE SOC intervention was designed to standardize the number of visits (3 antenatal and 12 postnatal visits) and the content of primary healthcare messages across treatment arms. Four of these visits promote exclusive breastfeeding (EBF) from birth to 6 months using modules designed to overcome contextual barriers to EBF [91, 92]. Other SOC modules include prevention of mother-to-child human immunodeficiency virus (HIV) transmission (PMTCT), antenatal care, hospital-based delivery, family planning, and immunizations.

### WASH Intervention

Within 6 weeks of enrollment (approximately 20 weeks’ gestation) into the WASH and WASH + IYCF arms of the trial (see companion article [85]), a Blair ventilated improved pit (VIP) latrine [93] is constructed at the participant's household and 2 “Tippy Tap” handwashing stations (locally manufactured, and adapting the model piloted by the Kenya WASH Benefits Trial [92]) are installed near the latrine and kitchen. WASH module 1 (delivered at 24 gestational weeks) and module 2 (at 32 gestational weeks) promote safe disposal of feces, and handwashing with soap after fecal contact and before food preparation and eating, respectively. Our intention was for the baby to be born into a household in which latrine use and household handwashing behaviors were normalized and habitual. WASH module 3 (protecting babies from fecal ingestion during explorative play) is delivered when the baby is 2 months old; a washable 2.8 × 3.0-m locally manufactured mat and plastic play yard (North States, Minneapolis, Minnesota) are provided at 2 months and 6 months, respectively. WASH module 4 (treat all drinking water given to babies after 6 months of EBF) is delivered at 4 months of age, along with point-of-use chlorination (WaterGuard: a dilute sodium hypochlorite solution, manufactured locally by Nelspot). Liquid soap and WaterGuard are regularly replenished from time of introduction (modules 2 and 5, respectively) until the infant is 18 months old. WASH module 5, delivered at 5 months of age, stresses the importance of freshly preparing or fully reheating all foods fed to infants. A review module is delivered at 12 months.

### IYCF Intervention

For the IYCF intervention (see companion paper [86]), IYCF module 1 (delivered at 5 months) links good infant feeding to child growth, health, and intelligence. IYCF module 2 (6 months) promotes feeding nutrient-dense food, including 20 g per day of the lipid-based nutrient supplement developed by the International Lipid-Based Nutrients Supplements Project [94], provided monthly when the baby is 6–18 months of age. Module 3 (7 months) is a participatory cooking demonstration in which any available household food is prepared and fed to the baby, stressing 3 messages from formative research [37]: (1) An infant can eat any food that an adult eats; (2) food should be ground so that the infant can swallow and digest it; (3) food that is locally available is important for the infant. Module 4 (8 months) promotes responsive feeding during illness, module 5 (9 months) promotes diet diversity, and a review module is delivered at 12 months.

## STUDY SITE AND ITS PREPARATION FOR THE TRIAL

### Study Site Selection and Description

The study site comprises the contiguous districts of Chirumanzu and Shurugwi (combined population of 158 821 in 2010) [95], 300 km south of Harare. Districts were chosen for SHINE based on the following criteria: predominantly rural, low sanitation coverage (53% for these districts, with 45% open defecation), high prevalence of child stunting (mean LAZ at 18 months and 24 months, −1.89 and −1.97, respectively), reasonable road access throughout the year, availability of primary healthcare services at the district level offering facility-based antenatal care and deliveries, geographical contiguousness, and stakeholder receptivity. There were no district-level data available on childhood anemia in the study areas; nationally, 74% of children aged 9–17 months were anemic in 2011, with no change from the previous 5-year period [96]. Among school-aged children, both study districts have a high prevalence of *Schistosoma hematobium* infection (23.5% in Chirumanzu, 55.9% in Shurugwi), but very low prevalence of soil-transmitted helminth infections (0% in Chirumanzu, 1.5% in Shurugwi) [97].

### Cluster Formation

The study area was divided into 212 clusters, defined as the catchment area of 1–4 VHWs. Digital maps of administrative boundaries of the study area were obtained from the Central Statistics Office and, using Google Earth, all homesteads and key landmarks (roads, clinics, schools, rivers) were plotted. Using large-scale maps, each of the 360 VHWs circled the houses they serve. Cluster-specific numbers of households, reproductive-age women, and <2-year-old children were gathered from VHW registers. Boundaries were finalized by grouping up to 4 VHWs into the same cluster if (1) they were working habitually as a team; (2) their catchment areas included homesteads within close proximity of each other; or (3) to achieve approximately the same number of households, pregnant women, and young children per cluster. About half of the clusters comprise the catchment area of a single VHW; on average, there are 1.6 VHWs per cluster.

### Cluster-Specific Sanitation and Water Surveys

In 2011–2012, we assessed cluster-specific water access and sanitation coverage so that these factors could be balanced across treatment arms during randomization (see companion article [98]). A georeferenced distribution dataset was constructed of all households in the 2 study districts using Google Earth satellite imagery. The spatial distribution of all water points was mapped through a comprehensive survey. Spatial analyses were conducted to calculate cluster-specific mean distance from households to the nearest functional perennial protected water source, and the proportion of households <500 m and >1500 m from such a water source. Sanitation facilities were surveyed in 13 households randomly selected from each cluster. The cluster-specific proportions of households with a VIP latrine, a VIP latrine less than one-half full, and a VIP latrine less than one-half full also accompanied by a nearby handwashing station were calculated. These cluster-specific water access and sanitation indicators were included as covariates in the constrained randomization process (see section 7).

### Community Engagement

Community engagement began 5 years prior to trial enrollment through discussion with leaders, strengthening of the PMTCT of HIV program, and VHW network strengthening. During a 3-month period before launching SHINE, we held 45 meetings throughout the study area, each attended by 50–75 influential community members and led by local government and district-level Zimbabwe MoHCC authorities. Following these consultations, Memoranda of Understanding were signed between each district, the MoHCC and Zvitambo, detailing SHINE activities and defining the tripartite roles.

### Hub Staffing and Facilities

We established 4 study hubs in the study area, strategically sited such that all households in the study area could be reached by motorbike in <2 hours from the nearest hub; all were established within MoHCC facilities. Each hub is staffed with approximately 25 people and includes specimen processing laboratories with −80°C archiving freezers, internet connection, offices, and warehousing.

## SAMPLE SIZE AND POWER

The enrollment goal was a total sample size of 4800 pregnant women, 1200 in each of the 4 trial arms, each of which is comprised of 53 clusters. The primary outcomes are LAZ and hemoglobin level at 18 months of age, stratified by maternal HIV status. The main trial inference will be based on outcomes of infants of HIV-negative women, of whom we expect at least 4080 based on an estimated 15% HIV prevalence among pregnant women at the time of designing the trial. Although the primary outcomes are continuous, the sample size is based on dichotomous versions of these, as we wanted to provide answers regarding stunting and anemia per se. Allowing for 20% loss of evaluable infants at 18 months (including pregnancy losses, infant mortality, and loss to follow-up), we expect about 816 measurements in each of the 4 study arms. With type I error of 5% (2-sided test), and power of 90%, and control group stunting (HAZ < −2) of 30%, we will be able to detect a reduction of about 8 percentage points (ie, from 30% to 22%) for the marginal effect of either of the interventions, even in the absence of an effect of the other intervention [99].

Calculations were based on an assumed coefficient of variation of the true proportion stunted at 18 months of 0.43 (derived from the 2011 Demographic Health Survey), and an effective loss of 33% of sample size due to cluster size variability, resulting in a design effect of 2.5. To be conservative, we used the same coefficient of variation for anemia. The largest design effect reported by Katz for stunting in several countries was 2.6 [100]. Using the design effect of 2.5, we have 89.7% power to detect a shift of 0.2 in weight-for-age and length-for-age *z* scores, assuming a within-randomized cluster variance of 1.25. For hemoglobin, assuming a standard deviation at 18 months of 128 g/L [101], we will be able to detect a shift of 26 g/L for either of the interventions with 95% power.

## RANDOMIZATION

**Figure 1.**
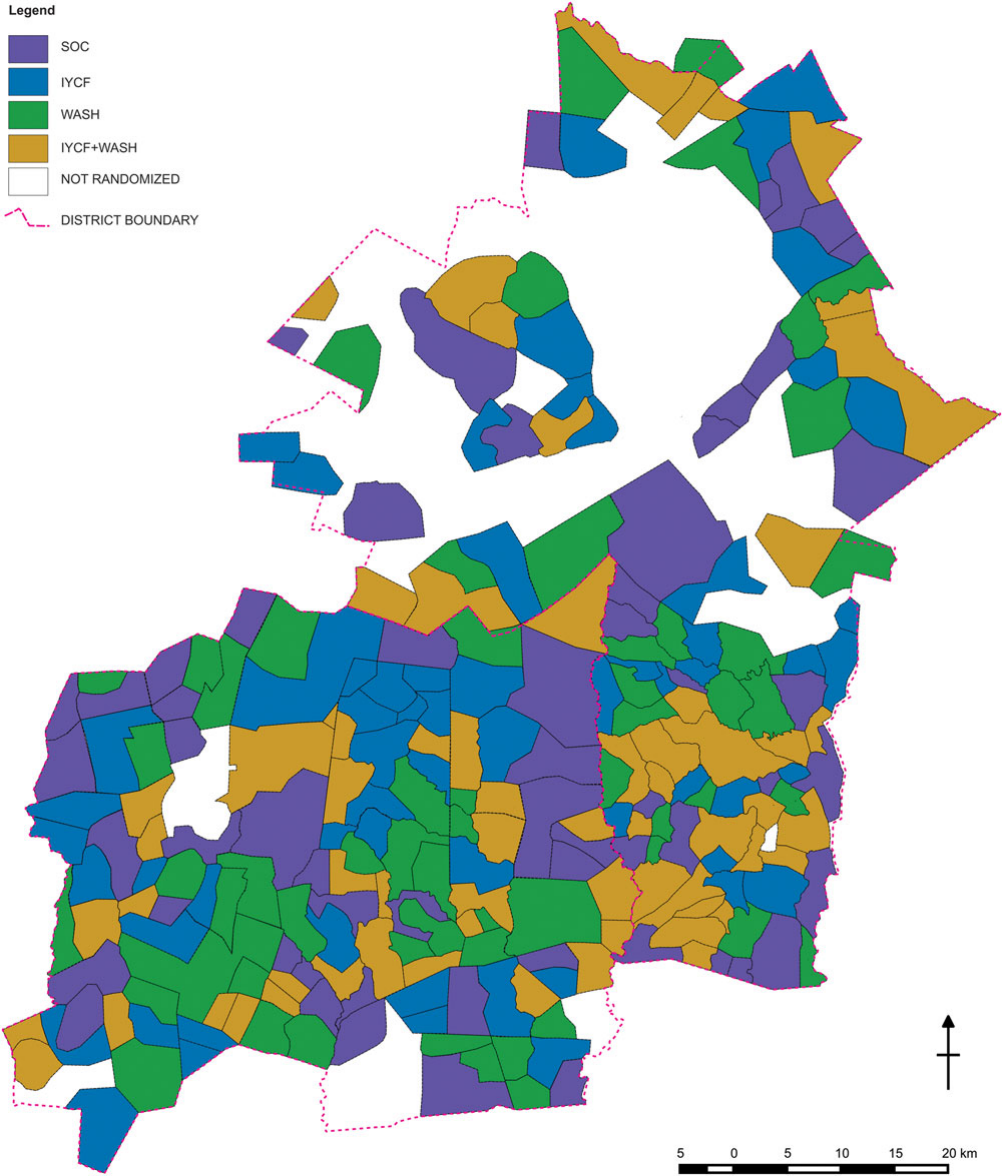
Randomized clusters of the Sanitation Hygiene Infant Nutrition Efficacy (SHINE) trial, Chirumanzu and Shurugwi districts, Zimbabwe. Uncolored areas were not randomized because at the time of mapping they were urban; commercial; not covered by a Ministry of Health and Child Care Village Health Worker; or uninhabited. Abbreviations: IYCF, infant and young child feeding; SOC, standard of-care; WASH, water and sanitation/hygiene.

A highly constrained randomization technique was used to allocate clusters (stratified by district) to treatments [102]. A GAUSS computer program generated millions of random permutations, enumerating those that met specified balance criteria for 14 variables related to geography, demography, water access, and sanitation coverage; a check was made for validity of the scheme on 5000 such acceptable allocations. Then, from the acceptable allocations that also met bias and validity specifications, 10 allocations were randomly selected. Each randomization scheme divided the 212 randomization units into 4 groups of approximately 53 units. Each scheme's corresponding color-coded map was printed on a separate sheet and displayed at a public forum attended by all elected councilors from the study area, district and provincial administrators, and MoHCC authorities. In their presence, 10 plastic balls (numbered 1–10) were placed in an opaque sack. A community representative selected one ball from the sack, thereby identifying which of the 10 numbered allocations would be used. Then, 4 balls (labeled A, B, C, and D) were placed in one sack, and 4 balls (labeled with the 4 treatment arms) were placed in a second sack. Representatives drew a ball from the first sack and a ball from the second sack, pairing a group of clusters with 1 of the 4 treatment arms, thereby mimicking a widely known World Cup draw procedure. This was repeated twice more to pair the next 2 groups of clusters with 2 more treatment arms. The remaining balls formed the final pairing. This second stage was included to provide an additional assurance of impartiality/randomness and a further opportunity for participation of the community leadership. The resulting map is shown in Figure 1, where the lines represent cluster boundaries and each color represents 1 of the 4 trial arms.

The randomization was stratified by district to achieve the same approximate number of clusters within each of the 4 study arms within each of the 2 districts. Although overall there were 53 clusters in each study arm, exact balance within each district was not possible, because 111 clusters were formed in one district and 101 in the other, neither of which was a multiple of 4. Forming appropriate clusters was deemed more important than achieving exact balance. The constraint criteria and the achieved values of the constraining variables by study arm are shown in Table 2 in the Supplementary Appendix.

## RECRUITMENT OF SUBJECTS

VHWs were trained to visit all 15- to 49-year-old women in their catchment area once every 5 weeks, to offer a urine pregnancy test to those who had missed a menses, and to refer pregnancies to antenatal clinic and (with the woman's assent) to SHINE. We enrolled all consenting pregnant women permanently residing in the rural parts of the study districts during the period of trial recruitment; women living in employer-provided housing or renting accommodation were excluded because their residence was unlikely to be permanent and they could not authorize construction of a latrine at the household. We strove to enroll women after gestational week 8 (to minimize miscarriage risk after enrollment) but before week 14 (to provide a substantial period for WASH households to take up optimal fecal disposal and handwashing behaviors before the baby was born).

## OUTCOME MEASUREMENT

### Research Visits

All research visits are conducted in study households by hub-based data collectors (DCs) who travel by motorcycle. Forty-three primary care nurses were recruited as DCs and given a 14-week training course in research ethics and Good Clinical Practice (GCP) (1 week); motorcycle riding (3 weeks); computing and data entry (2 weeks); HIV testing and counseling (3 weeks); SHINE questionnaire content and interviewing techniques (2 weeks); maternal and child anthropometry (1 week); clinical assessment and referral of infants according to Integrated Management of Childhood Illness [103] guidelines, and specimen collection and study procedure SOPs (2 weeks). Every 6 months, all DCs are standardized in maternal and infant anthropometry; a coefficient of reliability (R) value against a gold standard anthropometrist is calculated and DCs with R values <0.95 for intra- and interobserver accuracy are given further training and additional supervision in the field. Supervisors observe every DC conducting a research visit at least quarterly and conduct spot checks with study participants to evaluate DC performance.

Following referral of a pregnant woman to SHINE, a DC conducts a visit to confirm the woman's pregnancy (by repeat urine test) and eligibility for the trial, and to obtain written informed consent. Illiterate women provide a thumbprint in the presence of a witness. Subsequently, DCs make 2 antenatal visits, at approximately 10–20 weeks (baseline) and 32 weeks, and 5 postnatal visits when the infant is 1, 3, 6, 12, and 18 months of age (Table 3).

#### Maternal and Household Data

Maternal and household data collected at baseline include household composition, socioeconomic status, current WASH facilities and practices, and current IYCF practices if the household has a child <2 years old. The woman is interviewed regarding her antenatal care for the current pregnancy and recent morbidity; maternal capacities and depression are assessed (see companion article [82]). Information pertaining to maternal exposure to study messages and the quality of their interaction and relationship with the VHW is ascertained. (The collection of information on fidelity of intervention delivery is detailed in a companion article [81]). Maternal height, weight, mid-upper arm circumference, and blood pressure are measured; HIV status, hemoglobin, and urinalysis are tested by point-of-care methods; biospecimens are collected as described below (and in companion article [83] and Table 3; equipment used is listed in Table 3 in the Supplementary Appendix). Maternal and household factors are reassessed at subsequent research visits as detailed in Table 3.

#### Birth Information

Birth information including date, mode, complications, weight, length, and head circumference are transcribed from the institutional delivery register (or the “born before arrival” register for home deliveries, who generally present for birth certification within 2 days) and also from the mother's handheld antenatal care card. The same models (Table 3 of Supplementary Appendix 1) of infant scales and stadiometers used in trial research visits were installed by the trial in all 43 health institutions offering delivery services to women in the study area; anthropometry training is repeated every 6 months for health institution staff. For home deliveries, a visit is conducted to collect infant anthropometric measurements if SHINE learns of the delivery within 7 days. Birth dates are combined with last menstrual period date to calculate gestational age at birth and to categorize preterm birth.

#### Infant Data

Infant data are collected at ages 1, 3, 6, 12, and 18 months as detailed in Table 3. Infant weight, length, head circumference, and mid-upper arm circumference are measured at every postnatal visit. At 18 months of age, infant length (trial primary endpoint) is recorded 3 times. Equipment is calibrated weekly. Other infant information collected includes delivery details and birth outcomes; vaccination history; PMTCT care; 7-day morbidity history; and sick clinic and hospitalization history since last visit (eg, tuberculosis, malaria). Infant diarrhea (≥3 loose watery stools in 24 hours; or blood or mucus in stools) is assessed by 7-day recall and long-term recall (since prior research visit) in all infants at each postnatal visit, and by daily diary completion in a subgroup of infants (see EED substudy, section 10). Infant feeding knowledge and practice and household WASH knowledge and behaviors are reassessed by questionnaire and structured observation at every postnatal visit.

### Biospecimens Collected From All Trial Participants

At baseline, maternal stool and blood specimens are obtained; urine is collected over 2 hours following ingestion of an oral dose of lactulose and mannitol, to enable measurement of the urinary recovery of sugars by mass spectrometry and computation of the LM ratio, which provides a noninvasive indicator of intestinal absorptive capacity and permeability [104]. At 32 gestational weeks, maternal blood and stool are collected. At 18 months after delivery, blood is collected from all infants and mothers. Hemoglobin concentration is measured using the point-of-care HemoCue Hb 301 hemoglobinometer (HemoCue, Angelholm, Sweden) in all infants at 18 months of age (primary endpoint).

Samples are transported in cooler boxes by motorcycle to 1 of the 4 hub laboratories where they are processed and archived in −80°C freezers; every 2 weeks, samples are transferred from hub laboratories for long-term archiving in the Zvitambo Institute for Maternal and Child Health Research (Zvitambo) laboratory in Harare. Because both districts are known to have a high prevalence of schistosomiasis among school-aged children, women are tested for *Schistosoma haematobium* infection at baseline and at 32 weeks by urinary microscopy; women testing positive are referred for postnatal praziquantel treatment per MoHCC policy. Women are offered home HIV testing by DCs at baseline and 32 gestational weeks using a rapid test algorithm approved by the MoHCC and, if positive, urgently referred to antenatal clinics to access PMTCT interventions. Women who do not wish to know their HIV status have blood tested in the field laboratory using the same rapid test algorithm, but the result is not returned to them. Women are offered a repeat HIV test at 18 months postpartum to detect seroconversion (incidence <1% in Zimbabwe). HIV-infected women are advised to take their infants to the local clinic to undertake early infant diagnosis and commence cotrimoxazole prophylaxis at 6 weeks of age; blood samples from HIV-exposed infants collected from 3 months of age onward are additionally tested for HIV by DNA polymerase chain reaction in the Zvitambo laboratory and results returned to women with appropriate counseling, unless the mother does not wish to learn her infant's HIV status. In the case of maternal or infant deaths, a verbal autopsy is conducted with a family member and cause of death adjudicated by 2 study clinicians according to World Health Organization criteria [90].

## EED SUBSTUDY OF THE BIOLOGIC CAUSAL PATHWAY

A subsample of at least 1000 HIV-unexposed infants (250 per trial arm) and all consenting HIV-exposed infants are being recruited into the EED substudy (see companion article [85]). The substudy population comprises infants of women reaching their 32 weeks’ gestation visit from 1 May 2014 through the end of the trial. We will undertake analysis of the causal pathway separately for HIV-exposed and HIV-unexposed infants because maternal HIV infection and cotrimoxazole prophylaxis in HIV-exposed infants will likely modify the microbiota, intestinal inflammation, intercurrent infections, and systemic inflammation. HIV-unexposed infants are defined as those born to women who tested HIV-negative at baseline and 32 gestational weeks. For all dyads selected into the EED substudy, a paired maternal–infant stool and blood sample is collected at 1 month postpartum, and infant biospecimens are collected at 3, 6, 12, and 18 months of age to assess the EED pathway, as described elsewhere [83]. Mothers are asked to complete a daily morbidity diary using stickers to record episodes of infant illness (diarrhea; blood or mucus in stools; cough; fast or difficult breathing; fever; or lethargy interfering with feeding), for which they are sent a weekly cellphone reminder (to eliminate bias due to cellphone ownership, the trial provides a cellphone, solar charger, and airtime to mothers in the EED substudy). In a subgroup of 800 EED infants (all consenting EED infants reaching 3 months of age from 1 June 2015 to the end of the trial), additional specimens are collected during diarrheal episodes to evaluate the pathogens causing diarrhea in this setting using the TaqMan Array Card, and to compare pathogen-specific diarrhea rates across trial arms [105].

## OBSERVATIONAL STUDIES OF OTHER EXPOSURES AND OUTCOMES WITHIN SHINE

Several observational studies are being pursued within the SHINE study population, drawing upon the infrastructure, dataset, and sample archive. Maternal schistosomiasis (assessed by urine microscopy) and maternal and infant biomarkers of mycotoxin exposure (see companion article [106]) are being measured and will be analyzed in a longitudinal observational design as potential contributors to EED and inflammation.

We also will investigate the possibility that maternal EED and/or mycotoxin exposure during pregnancy contributes to adverse birth outcomes, including miscarriage, stillbirth, preterm delivery, fetal stunting, low birth weight, and neonatal death. Pregnancy losses are ascertained prospectively and data collected on risk factors and outcomes in a specific questionnaire. These studies are planned as case-control designs; the randomized interventions will be treated as covariates in analysis.

## DATA FLOW AND MANAGEMENT

DCs electronically collect questionnaire and observation data at all research visits using netbooks; questionnaires were programmed in Microsoft Access and SQL database and include quality control functions (eg, skip functions, permissible data range checks). Paper forms are available if technology fails in the field. At the close of each working day, all new data in the netbooks are copied onto 2 computers in the hub office: one accumulates all research data and the other is linked to the internet and automatically transfers data overnight to the main server in the Zvitambo office in Harare.

Blood, fecal, saliva, and urine samples are labeled with preprinted bar-code labels at the time of collection and transported to hub laboratories. After transfer to the Zvitambo laboratory in Harare, specimens are logged into laboratory information system prior to further processing, analysis, and archival.

## STATISTICAL ANALYSIS

### General Considerations

As a cluster-randomized trial, SHINE outcomes, in general, will be analyzed using statistical methods that account for within-cluster correlation. With a relatively large number of clusters (212) and for some analyses only a few events or prevalent cases per cluster, generalized estimating equations (GEEs) will be used, specifically the Stata GEE regression procedure, with exchangeable correlation structure within each cluster, for both continuous and discrete response variables. Primary analyses will contain 2 dummy variables representing each intervention; they will be unadjusted for other covariates. A log link function with Bernoulli response will be used for prevalence outcomes, to aid in interpretability by directly producing risk ratio estimates. Although the study is not powered to detect a statistical interaction effect between the 2 interventions, it will be estimated. If there is a statistically significant interaction, defined as *P* < .05 from a Wald test of the product term, we will also present results using a regression model with 3 terms to represent the 4 study arms.

In addition to these (unadjusted) primary analyses, we will report a set of adjusted estimates, conditional on baseline covariates described elsewhere [81]. The goals of such adjustment include accounting for baseline imbalances, reducing variance, and mitigating the effects of potential differential enrollment. We will also compare loss to follow-up across arms and conduct sensitivity analyses of such differential attrition [107]. We will test for effect modification by a limited number of hypothesized variables that might influence the effects of the randomized interventions in important ways. The main ones are sex of the child, and maternal HIV status (women testing HIV-positive at baseline or 32 gestational weeks vs no positive antenatal tests); women who seroconvert during the trial will be removed from the HIV-negative group in a sensitivity analysis. The theoretical basis for the additional hypotheses and our approach to assessment are described in companion papers [81, 82]. In the effect modification analyses, statistical significance will be assessed by multiplying the dummy variable for whichever intervention is being considered by the continuous/scale version of the potential modifier; the latter may then be categorized for communication purposes. For the EED substudy, questions that address the impact of the WASH intervention will use analyses that adjust for clustering, as per the GEE regression approach described above, and path analysis, as described in a companion article [83].

Reporting of results will follow the guidelines established in the extended CONSORT guidance for cluster-randomized trials [108].

### Analysis Populations

Most analyses, including the primary ones, are modified intent-to-treat at the child level. “Modified” refers to the requirement of an 18-month follow-up visit to determine the child's anthropometric status. Loss to follow-up is anything that prevents a child's 18-month visit from being completed, including pregnancy loss, infant death, refusal for further participation, and a permanent move outside of Zimbabwe. The analyses will be conducted according to the mother's assigned study arm based on her residence at the time of her enrollment, regardless of subsequent migration.

Secondary analyses may require varying levels of contact and/or adherence with a focus on the following modified per-protocol population [81]: 18-month length (and hemoglobin concentration) among those who participated in at least two-thirds (10 of 15) of the VHW visits, starting at 24 gestational weeks (visit defined as having contact at a scheduled visit, with 2 attempts made per visit).

## OVERSIGHT OF ETHICAL AND GCP COMPLIANCE

All research procedures are conducted in accordance with the Declaration of Helsinki and according to International Conference on Harmonisation GCP guidelines. The Medical Research Council of Zimbabwe and the Institutional Review Board of the Johns Hopkins Bloomberg School of Public Health provided initial and ongoing review and approval of the study protocol. Adverse events and serious adverse events are reported to both boards, according to their respective reporting requirements. An independent data safety and monitoring board (DSMB) was formed, comprising 2 physicians from the University of Zimbabwe and a statistician from the United Kingdom. The DSMB oversees implementation of the trial and monitors the safety and efficacy of interventions during quarterly reviews of interim data. A monitoring plan outlines the scope of internal and external monitoring for all trial activities. Internal monitoring is conducted quarterly at each field hub, and findings requiring corrective action are categorized as critical, major, or minor, with appropriate timelines for resolution. An external monitor appointed by the trial visits each hub every 6 months to conduct an inspection using his or her own monitoring and reporting tools.

## Supplementary Data

Supplementary materials are available at *Clinical Infectious Diseases* online (http://cid.oxfordjournals.org). Supplementary materials consist of data provided by the author that are published to benefit the reader. The posted materials are not copyedited. The contents of all supplementary data are the sole responsibility of the authors. Questions or messages regarding errors should be addressed to the author.

## APPENDIX

### SHINE TRIAL TEAM

Analysis and Writing Committee: Jean H. Humphrey, Andrew D. Jones, Amee Manges, Goldberg Mangwadu, John A. Maluccio, Mduduzi N. N. Mbuya, Lawrence H. Moulton, Robert Ntozini, Andrew J. Prendergast, Rebecca J. Stoltzfus, James M. Tielsch.

Technical and Management Team: Cynthia Chasokela, Ancikaria Chigumira, William Heylar, Preston Hwena, George Kembo, Florence D. Majo, Batsirai Mutasa, Kuda Mutasa, Philippa Rambanepasi, Virginia Sauramba, Naume V. Tavengwa, Franne Van Der Keilen, Chipo Zambezi.

Field Management Team: Dzivaidzo Chidhanguro, Dorcas Chigodora, Joseph F. Chipanga, Grace Gerema, Tawanda Magara, Mandava Mandava, Tafadzwa Mavhudzi, Clever Mazhanga, Grace Muzaradope, Marian T. Mwapaura, Simon Phiri, Alice Tengende.

#### Zvitambo Institute for Maternal and Child Health Research

Head Office: Cynthia Banda, Bernard Chasekwa, Leah Chidamba, Theodore Chidawanyika, Elisha Chikwindi, Lovemore K. Chingaona, Courage K. Chiorera, Adlight Dandadzi, Margaret Govha, Hlanai Gumbo, Karen T. Gwanzura, Sarudzai Kasaru, Rachel Makasi, Alois M. Matsika, Diana Maunze, Exevia Mazarura, Eddington Mpofu, Johnson Mushonga, Tafadzwa E. Mushore, Tracey Muzira, Netsai Nembaware, Sibongile Nkiwane, Penias Nyamwino, Sandra D. Rukobo, Thompson Runodamoto, Shepherd Seremwe, Pururudzai Simango, Joice Tome, Blessing Tsenesa.

#### Hub Teams

*Mvuma District Hospital Hub*: Umali Amadu, Beauty Bangira, Daniel Chiveza, Priscilla Hove, Horaiti A Jombe, Didymus Kujenga, Lenin Madhuyu, Prince Mandina-Makoni, Naume Maramba, Betty Maregere, Ellen Marumani, Elisha Masakadze, Phathisiwe Mazula, Caroline Munyanyi, Grace Musanhu, Raymond C. Mushanawani, Sibongile Mutsando, Felicia Nazare, Moses Nyarambi, Wellington Nzuda, Trylife Sigauke, Monica Solomon, Tendai Tavengwa.

*St Theresa's Hospital Hub*: Farisai Biri, Misheck Chafanza, Cloud Chaitezvi, Tsundukani Chauke, Collen Chidzomba, Tawanda Dadirai, Clemence Fundira, Athanasios C. Gambiza, Tatenda Godzongere, Maria Kuona, Tariro Mafuratidze, Idah Mapurisa, Tsitsi Mashedze, Nokuthula Moyo, Charles Musariri, Matambudzo Mushambadope, Tawanda R. Mutsonziwa, Augustine Muzondo, Rudo Mwareka, Juleika Nyamupfukudza, Baven Saidi, Tambudzai Sakuhwehwe, Gerald Sikalima, Jenneth Tembe.

*Shurugwi District Hospital Hub*: Tapiwanashe E. Chekera, Owen Chihombe, Muchaneta Chikombingo, Tichaona Chirinda, Admire Chivizhe, Ratidzai Hove, Rudo Kufa, Tatenda F. Machikopa, Wilbert Mandaza, Liberty Mandongwe, Farirai Manhiyo, Emmanuel Manyaga, Peter Mapuranga, Farai S. Matimba, Patience Matonhodze, Sarah Mhuri, Joice Mike, Bekezela Ncube, Walter T. S. Nderecha, Munyaradzi Noah, Charles Nyamadzawo, Jonathan Penda, Asinje Saidi, Sarudzai Shonhayi, Clemence Simon, Monica Tichagwa.

*Tongogara Clinic Hub*: Rachael Chamakono, Annie Chauke, Andrew F. Gatsi, Blessing Hwena, Hillary Jawi, Benjamin Kaisa, Sithembile Kamutanho, Tapiwa Kaswa, Paradhi Kayeruza, Juliet Lunga, Nomatter Magogo, Daniel Manyeruke, Patricia Mazani, Fungai Mhuriyengwe, Farisai Mlambo, Stephen Moyo, Tawanda Mpofu, Mishelle Mugava, Yvonne Mukungwa, Fungai Muroyiwa, Eddington Mushonga, Selestino Nyekete, Tendai Rinashe, Kundai Sibanda.

#### Ministry of Health and Child Care/Government of Zimbabwe

National, Provincial and District Offices: Milton Chemhuru, Jeffrey Chikunya, Vimbai F. Chikwavaire, Charity Chikwiriro, Anderson Chimusoro, Jotam Chinyama, Gerald Gwinji, Nokuthula Hoko-Sibanda, Rutendo Kandawasvika, Tendai Madzimure, Brian Maponga, Antonella Mapuranga, Joana Marembo, Luckmore Matsunge, Simbarashe Maunga, Mary Muchekeza, Monica Muti, Marvin Nyamana.

Village Health Workers

*Chirumanzu District*: Efa Azhuda, Urayai Bhoroma, Ailleen Biriyadi, Elizabeth Chafota, Angelline Chakwizira, Agness Chamhamiwa, Tavengwa Champion, Stella Chazuza, Beauty Chikwira, Chengeto Chingozho, Abigail Chitabwa, Annamary Dhurumba, Albert Furidzirai, Andrew Gandanga, Chipo Gukuta, Beauty Macheche, Bongani Marihwi, Barbara Masike, Eunice Mutangandura, Beatrice Mutodza, Angeline Mutsindikwa, Alice Mwale, Rebecca Ndhlovu, Norah Nduna, Cathrine Nyamandi, Elias Ruvata, Babra Sithole, Rofina Urayai, Bigboy Vengesa, Micheal Zorounye, Memory Bamule, Michael Bande, Kumbirai Chahuruva, Lilian Chidumba, Zvisinei Chigove, Kefas Chiguri, Susan Chikuni, Ruvarashe Chikwanda, Tarisai Chimbi, Micheal Chingozho, Olinia Chinhamo, Regina Chinokuramba, Chiratidzo Chinyoka, Xaviour Chipenzi, Raviro Chipute, Godfrey Chiribhani, Mary Chitsinga, Charles Chiwanga, Anamaria Chiza, Faith Chombe, Memory Denhere, Ephania Dhamba, Miriam Dhamba, Joyas Dube, Florence Dzimbanhete, Godfrey Dzingai, Sikhutele Fusira, Major Gonese, Johnson Gota, Kresencia Gumure, Phinias Gwaidza, Margret Gwangwava, Winnet Gwara, Melania Gwauya, Maidei Gwiba, Joyce Hamauswa, Sarah Hlasera, Eustina Hlukani, Joseph Hotera, Lovemore Jakwa, Gilbert Jangara, Micheal Janyure, Christopher Jari, Duvai Juru, Tabeth Kapuma, Paschalina Konzai, Moly Mabhodha, Susan Maburutse, Chipo Macheka, Tawanda Machigaya, Florence Machingauta, Eucaria Machokoto, Evelyn Madhumba, Learnard Madziise, Clipps Madziva, Mavis Madzivire, Mistake Mafukise, Marceline Maganga, Senzeni Maganga, Emmanuel Mageja, Miriam Mahanya, Evelyn Mahaso, Sanelisiwe Mahleka, Pauline Makanhiwa, Mavis Makarudze, Constant Makeche, Nickson Makopa, Ranganai Makumbe, Mascline Mandire, Eunice Mandiyanike, Eunice Mangena, Farai Mangiro, Alice Mangwadu, Tambudzai Mangwengwe, Juliet Manhidza, Farai Manhovo, Irene Manono, Shylet Mapako, Evangelista Mapfumo, Timothy Mapfumo, Jane Mapuka, Douglas Masama, Getrude Masenge, Margreth Mashasha, Veronica Mashivire, Moses Matunhu, Pazvichaenda Mavhoro, Godfrey Mawuka, Ireen Mazango, Netsai Mazhata, David Mazuva, Mary Mazuva, Filomina Mbinda, John Mborera, Upenyu Mfiri, Florence Mhandu, Chrispen Mhike, Tambudzai Mhike, Artwell Mhuka, Judith Midzi, Siqondeni Moyo, Michael Mpundu, Nicholas Msekiwa Msindo, Dominic Msindo, Choice Mtisi, Gladys Muchemwa, Nyadziso Mujere, Ellison Mukaro, Kilvera Muketiwa, Silvia Mungoi, Esline Munzava, Rosewita Muoki, Harugumi Mupura, Evelyn Murerwa, Clarieta Murisi, Letwin Muroyiwa, Musara Muruvi, Nelson Musemwa, Christina Mushure, Judith Mutero, Philipa Mutero, Patrick Mutumbu, Cleopatra Mutya, Lucia Muzanango, Martin Muzembi, Dorcus Muzungunye, Valeliah Mwazha, Thembeni Ncube, Takunda Ndava, Nomvuyo Ndlovu, Pauline Nehowa, Dorothy Ngara, Leonard Nguruve, Petronella Nhigo, Samukeliso Nkiwane, Luckson Nyanyai, Judith Nzombe, Evelyn Office, Beatrice Paul, Shambadzirai Pavari, Sylvia Ranganai, Stella Ratisai, Martha Rugara, Peter Rusere, Joyce Sakala, Prosper Sango, Sibancengani Shava, Margaret Shekede, Cornellious Shizha, Tedla Sibanda, Neria Tapambwa, John Tembo, Netsai Tinago, Violet Tinago, Theresa Toindepi, John Tovigepi, Modesta Tuhwe, Kundai Tumbo, Tinashe Zaranyika, Tongai Zaru, Kamurayi Zimidzi, Matilda Zindo, Maria Zindonda, Nyaradzai Zinhumwe, Loveness Zishiri, Emerly Ziyambi, James Zvinowanda.

*Shurugwi District*: Ekenia Bepete, Christine Chiwira, Naume Chuma, Abiegirl Fari, Samson Gavi, Violet Gunha, Fadzai Hakunandava, Constance Huku, Given Hungwe, Grace Maduke, Elliot Manyewe, Tecla Mapfumo, Innocent Marufu, Chenesai Mashiri, Shellie Mazenge, Euphrasia Mbinda, Abigail Mhuri, Charity Muguti, Lucy Munemo, Loveness Musindo, Laina Ngada, Dambudzo Nyembe, Rachel Taruvinga, Emma Tobaiwa, Selina Banda, Jesca Chaipa, Patricia Chakaza, Macdonald Chandigere, Annie Changunduma, Chenesai Chibi, Otilia Chidyagwai, Elika Chidza, Nora Chigatse, Lennard Chikoto, Vongai Chingware, Jaison Chinhamo, Marko Chinhoro, Answer Chiripamberi, Esther Chitavati, Rita Chitiga, Nancy Chivanga, Tracy Chivese, Flora Chizema, Sinikiwe Dera, Annacolleta Dhliwayo, Pauline Dhononga, Ennia Dimingo, Memory Dziyani, Tecla Fambi, Lylian Gambagamba, Sikangela Gandiyari, Charity Gomo, Sarah Gore, Jullin Gundani, Rosemary Gundani, Lazarus Gwarima, Cathrine Gwaringa, Samuel Gwenya, Rebecca Hamilton, Agnes Hlabano, Ennie Hofisi, Florence Hofisi, Stanley Hungwe, Sharai Hwacha, Aquiiline Hwara, Ruth Jogwe, Atanus Kanikani, Lydia Kuchicha, Mitshel Kutsira, Kumbulani Kuziyamisa, Mercy Kuziyamisa, Benjamin Kwangware, Portia Lozani, Joseph Mabuto, Vimbai Mabuto, Loveness Mabvurwa, Rebecca Machacha, Cresenzia Machaya, Roswitha Madembo, Susan Madya, Sheneterai Madzingira, Lloyd Mafa, Fungai Mafuta, Jane Mafuta, Alfred Mahara, Sarudzai Mahonye, Admire Maisva, Admire Makara, Margreth Makover, Ennie Mambongo, Murenga Mambure, Edith Mandizvidza, Gladys Mangena, Elliot Manjengwa, Julius Manomano, Maria Mapfumo, Alice Mapfurire, Letwin Maphosa, Jester Mapundo, Dorcas Mare, Farai Marecha, Selina Marecha, Christine Mashiri, Medina Masiya, Thembinkosi Masuku, Priviledge Masvimbo, Saliwe Matambo, Getrude Matarise, Loveness Matinanga, John Matizanadzo, Margret Maunganidze, Belinda Mawere, Chipiwa Mawire, Yulliana Mazvanya, Maudy Mbasera, Magret Mbono, Cynthia Mhakayakora, Nompumelelo Mhlanga, Bester Mhosva, Nomuhle Moyo, Over Moyo, Robert Moyo, Charity Mpakami, Rudo Mpedzisi, Elizabeth Mpofu, Estery Mpofu, Mavis Mtetwa, Juliet Muchakachi, Tsitsi Mudadada, Kudakwashe Mudzingwa, Mejury Mugwira, Tarsisio Mukarati, Anna Munana, Juliet Munazo, Otilia Munyeki, Patience Mupfeka, Gashirai Murangandi, Maria Muranganwa, Josphine Murenjekwa, Nothando Muringo, Tichafara Mushaninga, Florence Mutaja, Dorah Mutanha, Peregia Mutemeri, Beauty Mutero, Edina Muteya, Sophia Muvembi, Tandiwe Muzenda, Agnes Mwenjota, Sithembisiwe Ncube, Tendai Ndabambi, Nomsa Ndava, Elija Ndlovu, Eveln Nene, Enniah Ngazimbi, Atalia Ngwalati, Tafirenyika Nyama, Agnes Nzembe, Eunica Pabwaungana, Sekai Phiri, Ruwiza Pukuta, Melody Rambanapasi, Tambudzai Rera, Violet Samanga, Sinanzeni Shirichena, Chipiwa Shoko, More Shonhe, Cathrine Shuro, Juliah Sibanda, Edna Sibangani, Nikisi Sibangani, Norman Sibindi, Mercy Sitotombe, Pearson Siwawa, Magret Tagwirei, Pretty Taruvinga, Antony Tavagwisa, Esther Tete, Yeukai Tete, Elliot Thandiwe, Amonilla Tibugari, Stella Timothy, Rumbidzai Tongogara, Lancy Tshuma, Mirirayi Tsikira, Constance Tumba, Rumbidzayi Watinaye, Ethel Zhiradzango, Esther Zimunya, Leanmary Zinengwa, Magret Ziupfu, Job Ziyambe.

Student Investigators: James A. Church, Amy Desai, Dadirai Fundira, Ethan Gough, Rukundo A. Kambarami, Cynthia R. Matare, Thokozile R. Malaba, Tatenda Mupfudze, Francis Ngure, Laura E. Smith.

Collaborators: Sandy Cairncross, Val Curtis, Katherine L. Dickin, Jean-Pierre Habicht, Collen Masimirembwa, Peter Morgan, Gretel H. Pelto, Corinne Sheffner-Rogers, Roslyn Thelingwani, Paul Turner, Lindiwe Zungu.

Data and Safety Monitoring Board: Simon Cousens (Chair), Tariro Makadzange, Hilda A. Mujuru.
